# Highly Responsive and Self-Powered Photodetector Based on PtSe_2_/MoS_2_ Heterostructure

**DOI:** 10.3390/molecules29112553

**Published:** 2024-05-29

**Authors:** Haoran Li, Zhibin Yang

**Affiliations:** Key Laboratory of Optoelectronic Information and Technology, Ministry of Education, and College of Precision Instrument and Optoelectronics Engineering, Tianjin University, Tianjin 300072, China

**Keywords:** PtSe_2_, 2D heterostructure, high responsivity, self-powered

## Abstract

In recent years, 2D materials and their heterostructures have started to offer an ideal platform for high-performance photodetection devices. In this work, a highly responsive, self-powered photodetector based on PtSe_2_/MoS_2_ van der Waals heterostructure is demonstrated. The device achieves a noteworthy wide band spectral response from visible (405 nm) range to the near infrared region (980 nm). The remarkable photoresponsivity and external quantum efficiency up to 4.52 A/W, and 1880% are achieved, respectively, at 405 nm illumination with fast response time of 20 ms. In addition, the photodetector exhibits a decent photoresponsivity of 33.4 mA/W at zero bias, revealing the photodetector works well in the self-driven mode. Our work suggests that a PtSe_2_/MoS_2_ heterostructure could be a potential candidate for the high-performance photodetection applications.

## 1. Introduction

Photodetectors play an important role in diverse fields, including spectroscopy, communication, medicine and environmental monitoring [1,2]. Currently, commercial photodetectors with mature fabrication techniques are usually made by using group IV or III–V absorbers (Si, InSb, etc.) [3]. Silicon-based photodetectors are limited in their detection spectrum (up to ~900 nm) due to their large intrinsic bandgap (1.12 eV), while photodetectors relying on traditional compound semiconductors require costly fabrication processes or harsh operating conditions (e.g., low temperature). In addition, the low flexibility and high difficulty of small-size manufacturing limit the utilization of these materials in wearable and nanoscale optoelectronics. Recently, two-dimensional (2D) materials have demonstrated excellent potential for photodetection field thanks to the unique electronic and optoelectronic properties, which include high conductivity, layer-dependent bandgaps, outstanding light-matter interaction and high flexibility [4,5,6]. The non-existence of dangling bonds on the surface can reduce dark current from surface-recombination, which is beneficial for 2D photodetectors [7]. These excellent characteristics of 2D materials are beneficial to the application in optoelectronics [8,9]. 

Transition metal dichalcogenides (TMDs) have attracted numerous interests owing to the exceptional features, including high carrier mobility and adjustable bandgaps [10,11,12]. As one of the most representative materials among 2D chalcogen compounds, molybdenum disulfide (MoS_2_) presents outstanding electrical and optical properties. Both bulk MoS_2_ and multilayer MoS_2_ have indirect layer-dependent band gap in the range of 1.2–1.8 eV. When the thickness is reduced to monolayer, MoS_2_ has a direct band gap of 1.9 eV [13]. In 2017, by controlling the deviation of the atomic lattice, Ying et al. demonstrated a broadband photodetector based on MoS_2_ [14]. The photoresponsivity of the photodetection device is up to 50.7 mA/W at 445 nm, which is ascribed to the relatively large optical band gap of 2D MoS_2_ at room temperature. In addition to MoS_2_, PtSe_2_ is an important member of group-10 TMDs. The bandgaps of monolayer PtSe_2_ and bilayer PtSe_2_ are 1.2 eV and 0.21 eV, respectively. Moreover, when the number of layers reaches three and above, it exhibits semimetal characteristics [15], which enables it sensitive to near-infrared (NIR) region. Theoretically, the carrier mobility of few-layer PtSe_2_ at room temperature is as high as 4000 cm^2^/(Vꞏs) [16]. This feature gives PtSe_2_ the opportunity to be widely applied in optoelectronic devices. The relatively high dark current of 2D semimetal PtSe_2_ hamper the optoelectronic performance [17]. In addition, the weak light absorption of PtSe_2_ also hinders the performance of PtSe_2_-based photodetector [18]. Moreover, the inefficient separation of photogenerated carriers ascribed to the short carrier lifetime of semimetals increases the possibility of recombination and restrains the detectivity [19]. 

The interlayer van der Waals bonding of 2D materials enables the construction of heterostructure without consideration of lattice mismatch [12,13], which provides a suitable strategy to construct heterostructure that combines the advantages of different 2D materials. In order to circumvent the weaknesses of single 2D material, it is promising to construct 2D van der Waals heterostructures with different types [20]. Specifically, semimetal/semiconductor 2D heterostructure is widely used in the design of electronic and optoelectronic devices to achieve high-sensitivity photodetection [21,22]. The introduction of a 2D semiconductor can improve the detection performance. Kyoung et al. reported a near-infrared photodetector based on graphene/Ge heterostructure [23]. The barrier of the heterostructure was effectively regulated by the top gate, and the responsivity of the detector was as high as 0.75 A/W. Herein, designing semimetal/semiconductor heterostructure based on PtSe_2_ and MoS_2_ can realize suitable band alignment, which is essential for the performance. The built-in potential of the interface of semimetal/semiconductor PtSe_2_/MoS_2_ heterostructure can well suppress the dark current and promote the separation of carriers [24]. The PtSe_2_/MoS_2_ photodetector is expected to have excellent performance in wide band photodetection detection.

In this article, the semimetal/semiconductor PtSe_2_/MoS_2_ heterostructure was constructed by dry transfer process, which demonstrated a wide optical response from visible (405 nm) to NIR (980 nm) regions. The heterostructure was characterized by atomic force microscope (AFM) and Raman spectra, respectively. At room temperature, a good responsivity (4.52 A/W) has been obtained, which is much higher than other semimetal/semiconductor-based photodetectors. Furthermore, the PtSe_2_/MoS_2_ photodetector can work in self-driven mode, the photodetection performance was also studied. The results reveal the PtSe_2_/MoS_2_ heterostructure has great potential for high-performance wide detection range photodetection. 

## 2. Results and Discussion

**Figure 1:**
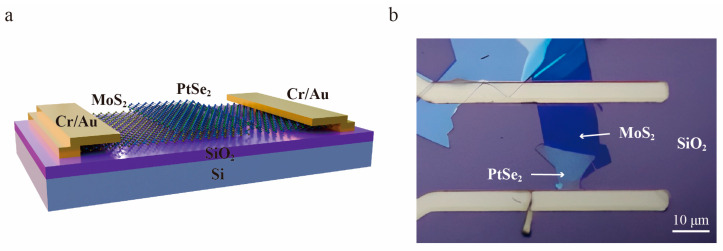
Structure of PtSe_2_/MoS_2_ heterostructure device. (**a**) Schematic diagram of the photodetector based on PtSe_2_/MoS_2_ heterostructure. (**b**) Optical image of PtSe_2_/MoS_2_ device.

Here, PtSe_2_/MoS_2_ heterostructure was fabricated by mechanical exfoliation and dry transfer method. Few-layer MoS_2_ and PtSe_2_ were subsequently transferred on the SiO_2_ (300 nm)/Si substrate to form an optimal heterostructure. The detailed process of heterostructure preparation is illustrated in the Materials and Methods section. Figure 1a,b depicts the schematic diagram of the photodetector based on PtSe_2_/MoS_2_ heterostructure and an optical image of the device, respectively. The dark blue portion of the heterostructure is 2D MoS_2_, while the light gray portion belongs to 2D PtSe_2_. In addition, The Au/Cr (80 nm/15 nm) electrodes of the device were prepared by standard photolithography and thermal evaporation. Herein, given the weak adhesion of the Au, Cr layer was used to improve the adhesion between electrodes and substrate. In addition, the channel width of the heterostructure device is 50 μm. Conversely, the channel length is 20 μm. As depicted in Figure 1b, the PtSe_2_ layer was superimposed on the MoS_2_ layer, which allows the overlapping area to be determined based on the contrast of colors. In addition, the area of the overlapping region can be estimated by calculating the area of a triangle. For an estimate based on the scale, the overlapping area is approximately 50 μm^2^. Owing to the 2D PtSe_2_ having wider absorption spectra than that of few-layer MoS_2_, the PtSe_2_ layer was transferred to the top of the heterostructure. 

**Figure 2:**
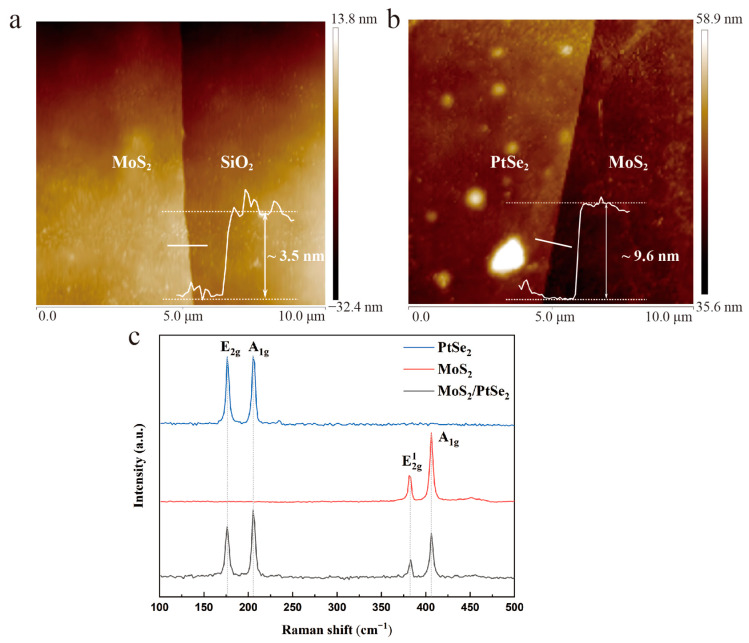
Raman spectra and AFM measurements of PtSe_2_/MoS_2_ heterostructure. (**a**) Raman spectra of MoS_2_, PtSe_2_ and PtSe_2_/MoS_2_ heterostructure, respectively (**b**,**c**) AFM measurements of MoS_2_ and PtSe_2_, respectively.

To precisely evaluate the thickness and morphology of PtSe_2_/MoS_2_ heterostructure, it was measured by AFM. Herein, the thickness of MoS_2_ is estimated to be 3.5 nm, and PtSe_2_ is approximately 9.6 nm, respectively (Figure 2a,b). Monolayer MoS_2_ and PtSe_2_ have thicknesses of roughly 0.65 nm and 0.88 nm, respectively [19,25]. Thus, the number of layers of MoS_2_ is approximately five, while the number of PtSe_2_ layers is approximately 11. Furthermore, the Raman spectra of separated PtSe_2_, separated MoS_2,_ and PtSe_2_/MoS_2_ heterostructure were measured. The wavelength of the used laser is 532 nm. As depicted in Figure 2c, in PtSe_2_ region, the two characteristic peaks of the Raman spectrum of E_2g_ and A_1g_ were located at 176.3 cm^−1^ and 205.4 cm^−1^, respectively, which belong to the E_g_ in-plane vibration mode and A_1g_ out-of-plane vibration mode of the Se atom, respectively. The peak intensity of E_2g_ and A_1g_ is similar. This phenomenon proves that the number of PtSe_2_ layer is approximately 10 [26]. Moreover, for 2D MoS_2_, the in-plane vibration mode $E_{2g}^{1}$ and out-of-plane vibration A_1g_ mode were located at 381.5 cm^−1^ and 406.1 cm^−1^, respectively. The measured energy difference of MoS_2_ can also be used to estimate the layer number is approximately five, which is consistent with the AFM results [25]. Both results of PtSe_2_ and MoS_2_ are consistent with the previous reports [27,28], indicating that few-layer PtSe_2_ and MoS_2_ nanosheets were mechanical exfoliated. In addition, these peaks all appear in the Raman spectra in the overlapping region of the heterostructure without obvious peak shift, confirming the formation of a good-quality van der Waals heterostructure after the fabrication process. The intensity of the measured Raman characteristic peak in the heterostructure region decreases compared with that of a single material, which is ascribed to the interlayer coupling within the overlapping area of different materials [29]. 

**Figure 3:**
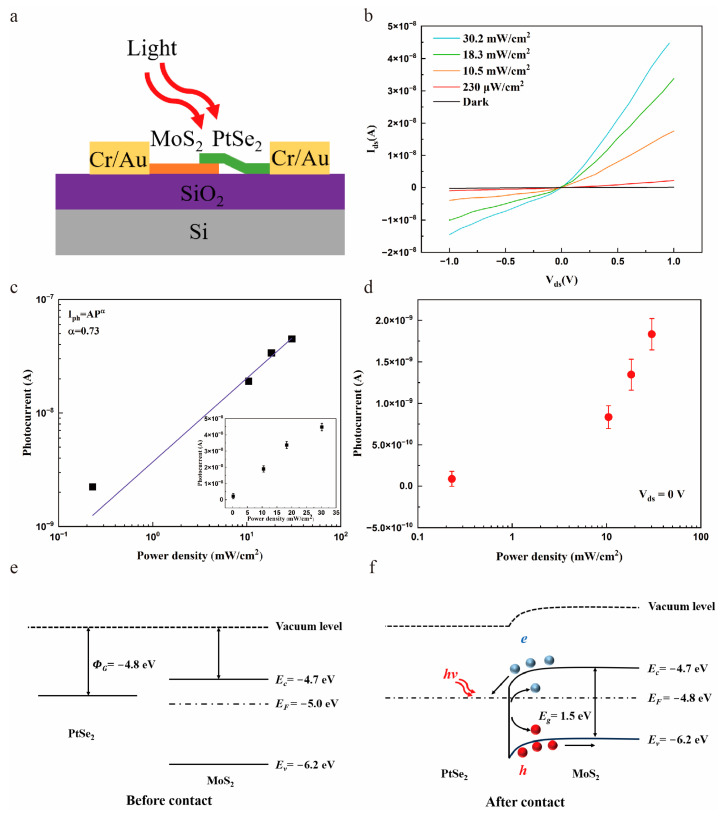
Photoresponse characteristics and band structure of the photodetector based on PtSe_2_/MoS_2_ heterostructure. (**a**) Schematic illustration of photodetector under 405 nm light emitting diode illumination. (**b**) Under different intensities of 405 nm and dark conditions, *I*_ds_-*V*_ds_ characteristic of photodetector based on PtSe_2_/MoS_2_ heterostructure. (**c**) Photocurrent of the PtSe_2_/MoS_2_ device under different power densities (*V*_ds_ = 1 V, λ = 405 nm). The inset *I*-*P* figure with the error bar indicates the device was measured multiple times under the same condition. (**d**) Under zero bias, dark current and the irradiation -power-dependent photocurrent under 405 nm irradiation. (**e**,**f**) Band structure of PtSe_2_/MoS_2_ heterostructure before and after contact.

To assess the optoelectronic properties of the PtSe_2_/MoS_2_ device, further characterization was implemented under the illumination of different wavelengths (405 nm, 700 nm and 980 nm). Without specific description, all the experiments were conducted on the photodetector portrayed in Figure 1b. Herein, the measured current of the photodetector shows a strong dependence on the power density. Figure 3b illustrates the *I*_ds_-*V*_ds_ characteristic of PtSe_2_/MoS_2_ photodetector under conditions of darkness and 405 nm light illumination with different power densities (230 μW/cm^2^–30.2 mW/cm^2^). (The *I*_ds_-*V*_ds_ characteristic under illumination of 700 nm and 980 nm can be found in Figure S1). A nonlinear *I*_ds_-*V*_ds_ characteristic was observed which ascribed to the good contact of the interface of the heterostructure. The *I*_ds_ under forward bias is much larger than that under reverse bias. Under the illumination of different power densities (230 μW/cm^2^, 18.3 mW/cm^2^, 10.5 mW/cm^2^ and 30.2 mW/cm^2^) of the illumination, *I*_ds_ of the heterostructure reaches the value of 4.48 × 10^−8^ A, 3.37 × 10^−8^ A, 1.75 × 10^−8^ A and 2.23 × 10^−9^ A, respectively, with applied external bias of 1 V. In addition, the dark current of the device is as low as 1.7 × 10^−10^ A (*V*_ds_ = 1 V), which is approximately four orders of magnitude smaller than that of PtSe_2_-based photodetector [18]. The reason for the low dark current is that the barrier at the interface of heterostructure limits the carrier drift. As the power density of illumination increases, the generated carriers increase significantly, and the photocurrent increases correspondingly. This phenomenon demonstrates that the measured photocurrent has a strong dependence on light intensity. The measured photocurrent also shows a similar trend under different power densities of 700 nm and 980 nm illumination. Based on the recorded experimental data, to systematically study the photoresponse of PtSe_2_/MoS_2_ photodetector, the net photocurrent (*I*_ph_ = *I*_ds_ − *I*_dark_) was extracted at *V*_ds_ = 1 V, which is dependent on the illumination power density, where *I*_dark_ stands for the dark current of the device. As depicted in Figure 3c, *I*_ph_ exhibits a sublinear increasing trend which able to be illustrated by the power law formula of:

(1) $$I=AP^{\alpha }$$

where *A* is a constant, *P* represents the incident power density and *α* represents the power law index, which is closely related to the quality of the photodetector. When *α* = 0 and the photogating effect determines the generation of photocurrent [30]. Conversely, when *α* = 1, the photoconductivity effect becomes the main mechanism of photocurrent generation. The value of *α* is usually used to analyze the mechanism of photocurrent generation. Herein, *α* can be extracted as 0.73, 0.68, and 0.66 for 405 nm, 700 nm and 980 nm, respectively (Figure S2). Furthermore, the value of α deviating from 1 means the loss of photogenerated carriers. Therefore, such a power law behavior indicates there are some trap states exist at the interface of PtSe_2_ and MoS_2_. Therefore, the photocurrent generation of the device was determined by these two effects. With the increasing power density, more photogenerated carriers will fill in the trap states, which can effectively enhance the possibility of photocarrier recombination and decrease the lifetime of carriers (τ). The saturation of the photocurrent leads to the lower value of the power law index α because of this. An analogous *α* variation trend was previously observed in other photodetectors based on 2D heterostructures [31]. 

As a new type of the state-of-art photodetection device, self-powered photodetectors have begun to receive more and more attention [32]. The ability to realize photodetection without external power supplies increases the possibility of applications in complex environments. Given this, we demonstrate that the PtSe_2_/MoS_2_ heterostructure can operate in self-driven mode. The photoresponse characteristic of the self-driven PtSe_2_/MoS_2_ heterostructure was also investigated. Under zero bias, Figure 3d plots the *I*_ds_-*V*_ds_ under 405 nm light irradiation ranging from 230 μW/cm^2^ to 30.2 mW/cm^2^. When the power density increases, photocurrent increased gradually, indicating the PtSe_2_/MoS_2_ device able to operate in self-driven mode [33]. When the power density is 230 μW/cm^2^, the photocurrent can reach 8.87 × 10^−11^ A. The ability of the PtSe_2_/MoS_2_ heterostructure to detect weak signals without external bias highlighting the application potential in optical communication. It is also worth noting that the device was tested multiple times under the same measurement conditions. As shown in Figure 3d, the calculated error bar clearly indicates that the detector can work stably. 

To better understand the operating mechanism of PtSe_2_/MoS_2_ photodetector, the band diagrams of the two different materials before and after contact were drawn based on their characteristics [27,34]. As shown in Figure 3e, the 11-layer PtSe_2_ can be regarded as a semimetal [31]. The Fermi level of the multi-layer PtSe_2_ is approximately 0.2 eV higher than that of MoS_2_ [27,34]. Therefore, when multi-layer PtSe_2_ is in contact with few-layer MoS_2_, electrons flow from PtSe_2_ to MoS_2_, and holes flow toward opposite directions (Figure 3f). Eventually, a strong built-in electric field is formed at the interface from PtSe_2_ to MoS_2_ when the heterostructure reaches equilibrium, resulting in the obvious band-bending. This formed barrier can efficiently suppress the dark current, which is beneficial to obtaining a higher net photocurrent. When the incident light irradiates the surface of the material, electron-hole pairs are generated and separated by the built-in potential then collected by electrodes to increase photocurrent significantly. Furthermore, the external electric field can effectively promote the separation of carriers in the reverse biased photodetector, which increases the photocurrent significantly. Therefore, the net photocurrent of the PtSe_2_/MoS_2_ heterostructure is much larger than the dark current, which gives it a decent photodetection capability.

**Figure 4:**
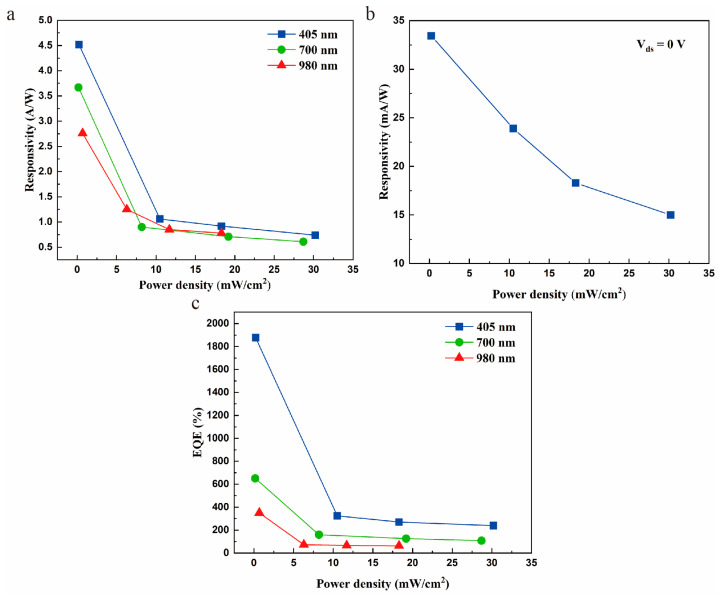
Performance of the PtSe_2_/MoS_2_ device. (**a**) Responsivity as a function of illumination intensities with different wavelengths. The eternal bias (*V*_ds_) was 1 V. (**b**) Responsivity as a function of self-driven PtSe_2_/MoS_2_ photodetector for 405 nm. (**c**) Under illumination of different wavelengths, the variation of EQE with power density. The eternal bias (*V*_ds_) was 1 V.

Some important figure-of-merits (FOMs) of the PtSe_2_/MoS_2_ photodetector can be calculated using the measured *I*_ph_. Photoresponsivity (*R_λ_*) plays a significant role in determine the performance of photodetectors and the expression is:

(2) $$R=I_{ph}/PS$$

where *I*_ph_ is the net photocurrent, *P* is the power density of the illumination with different wavelengths, and *S* denotes the effective responsive region. Photoresponsivity precisely reflects the merits of the photoelectric conversion efficiency of the photodetection device. Therefore, it is essential to study the responsivity of the device. Based on Equation (2), the responsivity of PtSe_2_/MoS_2_ heterostructure was calculated to be 4.52 A/W for 405 nm illumination with power density of 230 μW/cm^2^, which is approximately hundreds of times higher than that of MoS_2_/CdTe heterostructure based photodetector (10 mA/W, 405 nm) [35]. Under the same eternal bias (*V*_ds_ = 1 V), the responsivity of the device at 700 nm was approximately 3.67 A/W. Moreover, when the detection wavelength is up to NIR (980 nm), the responsivity of the device is still able to reach 2.76 A/W. This value of the heterostructure is significantly higher than that of a device based on PtSe_2_/CdTe heterostructure [36]. These results demonstrate the PtSe_2_/MoS_2_ device is highly sensitive to visible and NIR illumination. The main reason for the high responsivity is the built-in potential at the interface of PtSe_2_ and MoS_2_ can effectively promote carrier separation. Moreover, the high carrier mobility of PtSe_2_ is also valuable for decent responsivity. In addition, as depicted in Figure 4a, the responsivity of the PtSe_2_/MoS_2_ photodetector decreases with increasing incident power density. This finding indicates that the trap states capture the photogenerated carriers under low power intensity, which leads to a reduction of carrier recombination possibility [37]. Meanwhile, it also demonstrates that lower proportion of photogenerated carriers are collected at high light power intensity [38]. The generated carriers may screen the internal field could be another reason for the relationship of responsivity on light intensity [39]. 

Conversely, detectivity (D*) is an important FOM to evaluate the sensitivity of photodetectors. The value of detectivity can be calculated by equation:

(3) $$D^{*}=\frac{R_{\lambda }S^{\frac{1}{2}}}{{\left(2eI_{dark}\right)}^{\frac{1}{2}}}$$

where *R_λ_* is the photoresponsivity, *S* represents the effective portion of the heterostructure, *e* represents the electronic charge and *I*_dark_ is the dark current, respectively. The detectivity of PtSe_2_/MoS_2_ heterostructure was as high as 9.24 × 10^11^ Jones at 405 nm. Furthermore, the detectivity at 700 nm and 980 nm can achieve up to 7.5 × 10^11^ Jones and 5.63 × 10^11^ Jones, respectively. The detectivity of the device is outperformed than that of single MoS_2_-based photodetector [40]. For the purpose of accurately calculating the detectivity of the photodetector, the noise of the device should be systemically examined and studied under different frequencies [41]. As a future work, further measurements will be comprehensively carried out to characterize the noise current of the device. The noise-related detectivity under different frequencies will be accurately measured as well.

In addition, external quantum efficiency (*EQE*) is regarded as a critical FOM of the photodetector, which is the ratio between the generated electron-hole pair per second and the number of incident photons per second. *EQE* characterizes the efficiency of a photodetector in converting photons into electrons. In addition, *EQE* can be obtained by equation:

(4) EQE=Rhc/eλ

where *h* is the Planck constant and *c* is the speed of light. As depicted in Figure 4c, *EQE* exhibits a similar varying trend with the power density as the responsivity, reaching the highest value of 1880%, 641% and 349% for 405 nm, 700nm and 980 nm, respectively. The high *EQE* is much greater than the photodetector based on WSe_2_/SnSe_2_ heterostructure [42]. Here, *EQE* is higher than 100% indicating a large gain in the photodetector, which may attributed to the photogenerated carriers recirculated multiple times before reaching the electrodes [43].

In addition, we also calculated and analyzed the critical FOMs of the PtSe_2_/MoS_2_ photodetector which operates in self-driven mode. The responsivity of the self-driven PtSe_2_/MoS_2_ device is up to 33.4 mA/W at 405 nm (Figure 4b), which outperforms than that of the MoS_2_/WS_2_ heterostructure based self-driven photodetector [44]. The characteristic of working without external bias gives the heterostructure fine application prospects in the field of highly sensitive detections. Furthermore, under 405 nm illumination, the EQE of the self-driven PtSe_2_/MoS_2_ heterostructure is still 139%. 

**Figure 5:**
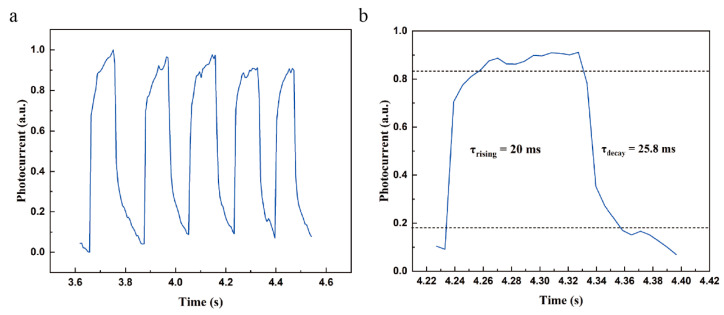
Time-resolved photoresponse of PtSe_2_/MoS_2_ photodetector. (**a**) Photoswitching behavior of PtSe_2_/MoS_2_ photodetector results from the irradiation of 405 nm. (**b**) Photoresponse of the PtSe_2_/MoS_2_ photodetector to a single pulse of incident light. The rise time of the device is 20 ms and the decay time is 25.8 ms.

Response time is another fundamental FOM which determines the maximum operating frequency of a photodetector. In previous studies, the response time of 2D heterostructure-based photodetectors is able to outperform than that of a single 2D material-based device [45]. The response of PtSe_2_/MoS_2_ photodetector was measured under the illumination of 405 nm wavelength with 30.2 mW/cm^2^ power density, which was switched between on and off states on a regular basis. As shown in Figure 5a, the time-dependent photoresponse maintains excellent stability and reliability after several switching cycles. Generally, rise time (τ_rising_) and decay time (τ_decay_) can be defined by the amount of changing time from 10%/90% to 90%/10% of net photocurrent. Herein, a photocurrent within a switching cycle is selected for analysis (Figure 5b). The measured τ_rising_ and τ_decay_ are 20 ms and 25.8 ms, respectively. The performance is comparable to those photodetectors based on mechanical exfoliated and transferred van der Waals heterostructure [46,47]. The steep edge of both the rise and decay time clearly indicates that the photogenerated carriers are able to separate promptly. The response time is limited by the phenomenon that carriers are trapped during the transport from the heterostructure to the metal contacts [47]. The response time can be improved by designing suitable patterned electrodes to facilitate the collection of photogenerated carriers. In addition, fabricating heterostructure with clean interface or reducing electrode spacing are also optimal methods to shorten response time [40]. All these results indicate that the PtSe_2_/MoS_2_ heterostructure is important for the development of state-of-art image processing and optical communications. 

## 3. Materials and Methods

### 3.1. Fabrication of PtSe_2_/MoS_2_ Heterostructure Photodetector

**Figure 6:**
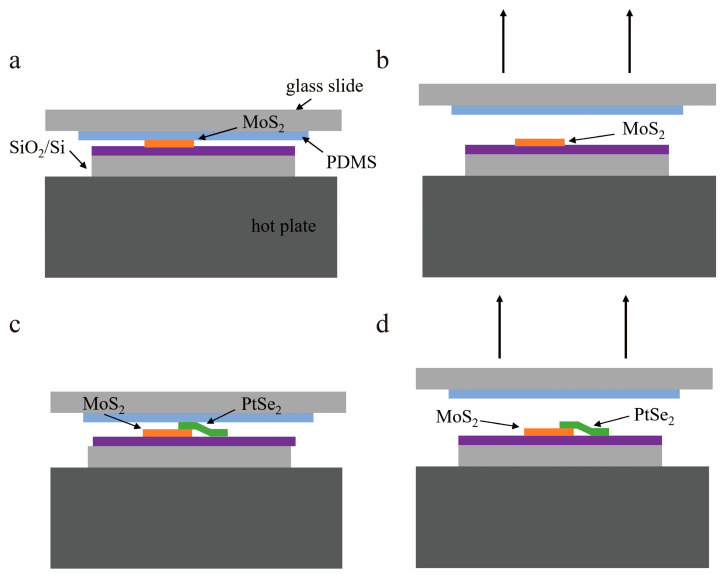
Fabrication process of PtSe_2_/MoS_2_ heterostructure. (**a**) The PDMS film with MoS_2_ was pressed onto the substrate. (**b**) The PDMS was elevated after successful transfer. (**c**) The PDMS film with PtSe_2_ was tightly pressed to the pre-transferred MoS_2_ substrate. (**d**) The PDMS was elevated after the second transfer process.

Figure 6 illustrates a detailed fabrication process of PtSe_2_/MoS_2_ heterostructure. Bulk PtSe_2_ and MoS_2_ were purchased from Nanjing MKNANO Tech. Co. Ltd. (Nanjing, China). The 2D PtSe_2_/MoS_2_ heterostructure was prepared by dry transfer with the assistance of polydimethylsiloxane (PDMS). First, the few layer 2D MoS_2_ was prepared by mechanical exfoliation. The tape with MoS_2_ was then tightly pasted with the PDMS film, pressed the tape with gentle pressure, and the MoS_2_ was transferred to PDMS completely. As shown in Figure 6a,b, the PDMS with 2D MoS_2_ stamped onto SiO_2_ (300 nm)/Si substrate and heated up to 80 °C for 5 min, then 2D MoS_2_ can be completely transferred to the substrate. After that, repeating a similar process (Figure 6c,d), the PtSe_2_ layer was stacked on 2D MoS_2_ when the substrate was heated up to 100 degrees. With the assistance of an optical microscope, the overlapping region of the PtSe_2_/MoS_2_ heterostructure can be precisely controlled. After spin-coating the S1813 photoresist on the substrate at the speed of 6000 rpm for 30 s, it was baked at 100 degrees for 1 min. Then, the designed electrodes were patterned on the PtSe_2_/MoS_2_ heterostructure by photolithography. The Cr (15 nm)/Au (80 nm) was deposited on the heterostructure by thermal evaporation, then followed by standard lift-off process to remove the undesired photoresist. 

### 3.2. Characterization of PtSe_2_/MoS_2_ Heterostructure

The morphologies structure of the PtSe_2_/MoS_2_ heterostructure was characterized by an optical microscope whose model is WITec, alpha 300R (Oxford Instruments, Abingdon, UK). The Raman spectra were studied by using a Raman spectrometer (model: HORIBA JOBIN YVON, HR800, Horiba, Kyoto, Japan), with a 532 nm laser source. Bruker Dimension Icon AFM (Bruker, Billerica, MA, USA) was used to study the thickness of 2D MoS_2_ and 2D PtSe_2_. A micromanipulator probe station SM-4 with a Keithley 2450 (Tektronix, Hongkong, China) source meter was used to investigate the optoelectronic properties of PtSe_2_/MoS_2_ photodetector. The light source used in experiments were light emitting diodes at 405 nm, 700 nm and 980 nm. Every measurement was carried out at room temperature in ambient conditions.

## 4. Conclusions

In summary, a high-performance photodetector based on PtSe_2_/MoS_2_ heterostructure has been designed and successfully fabricated. The characteristics of the photodetector were fully investigated. The device has demonstrated a wide range of photodetection from visible (405 nm) to NIR (980 nm). Furthermore, the photodetector exhibits many excellent FOMs including high responsivity (4.52 A/W for 405 nm, 3.67 A/W for 700 nm, 2.76 A/W for 980 nm); high EQE (1880% for 405 nm, 641% for 700nm and 349% for 980 nm); and fast response time (20 ms/25.8 ms), which makes it promising for application in high-performance optoelectronics. In addition, the detector has been found able to work in self-driven mode. Under 405 nm illumination, the responsivity and EQE of the photodetector operating in the self-driven mode can reach 33.4 mA/W and 139%, respectively. Such properties of PtSe_2_/MoS_2_ photodetector demonstrated in this work pave a new way for the development of high-performance photodetection applications. In future work, the detecting range of the heterostructure device ought to be further expanded to meet the requirements of modern science and technology for broadband detection. Additionally, the large-scale synthesis of PtSe_2_/MoS_2_ heterostructure also needs to be investigated. Furthermore, in order to achieve better application in practice, arrayed photodetectors and imaging devices will be developed as well.

## Data Availability

Data are contained within the article and Supplementary Materials.

## Supplementary Materials

The following supporting information can be downloaded at: https://www.mdpi.com/article/10.3390/molecules29112553/s1, Figure S1: *I*_ds_-*V*_ds_ curve of PtSe_2_/MoS_2_ photodetector under 700 nm and 980 nm irradiation; Figure S2: Photocurrent as a function of power density under 700 nm and 980 nm irradiation.
