# Supplementary material for: Highly Responsive and Self-Powered Photodetector Based on PtSe2/MoS2 Heterostructure
Source: Molecules. 2024 May 29;29(11):2553. doi: 10.3390/molecules29112553 (PMC11173480; doi:10.3390/molecules29112553)
Supplement: Supplementary file 1 [file molecules-29-02553-s001.zip › molecules-2958688-supplementary.pdf]

# Supplementary Materials

## Highly Responsive, Broadband and Self-Powered Photodetector Based on $PtSe_2/MoS_2$ Heterostructure

**Haoran Li [1], Zhibin Yang ***

Key Laboratory of Optoelectronic Information and Technology, Ministry of Education, and College of Precision Instrument and Optoelectronics Engineering, Tianjin University, Tianjin 300072, China

* Correspondence: zbyang0417@tju.edu.cn

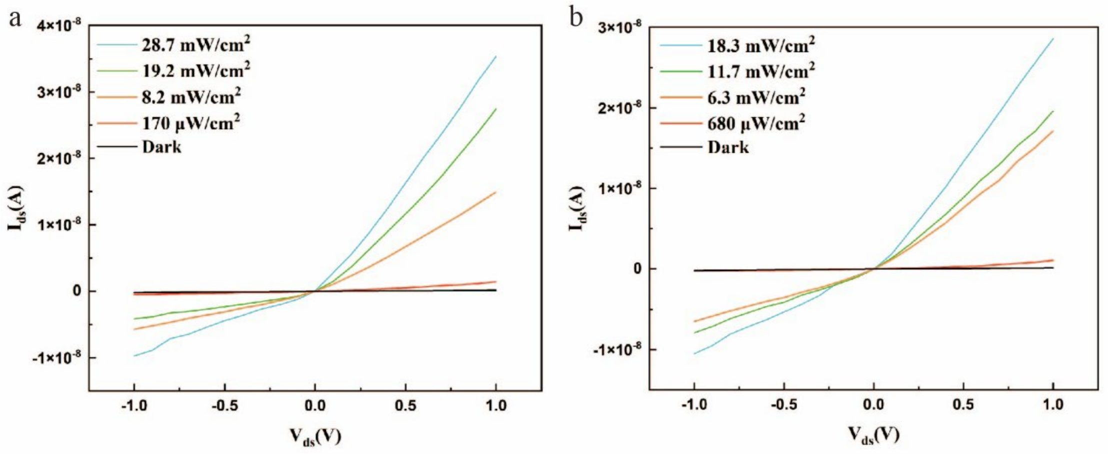


Figure S1. (a, b) The curves of $I_{ds}$-$V_{ds}$ for $PtSe_2/MoS_2$ photodetector illuminated under 700 and 980 nm, respectively.

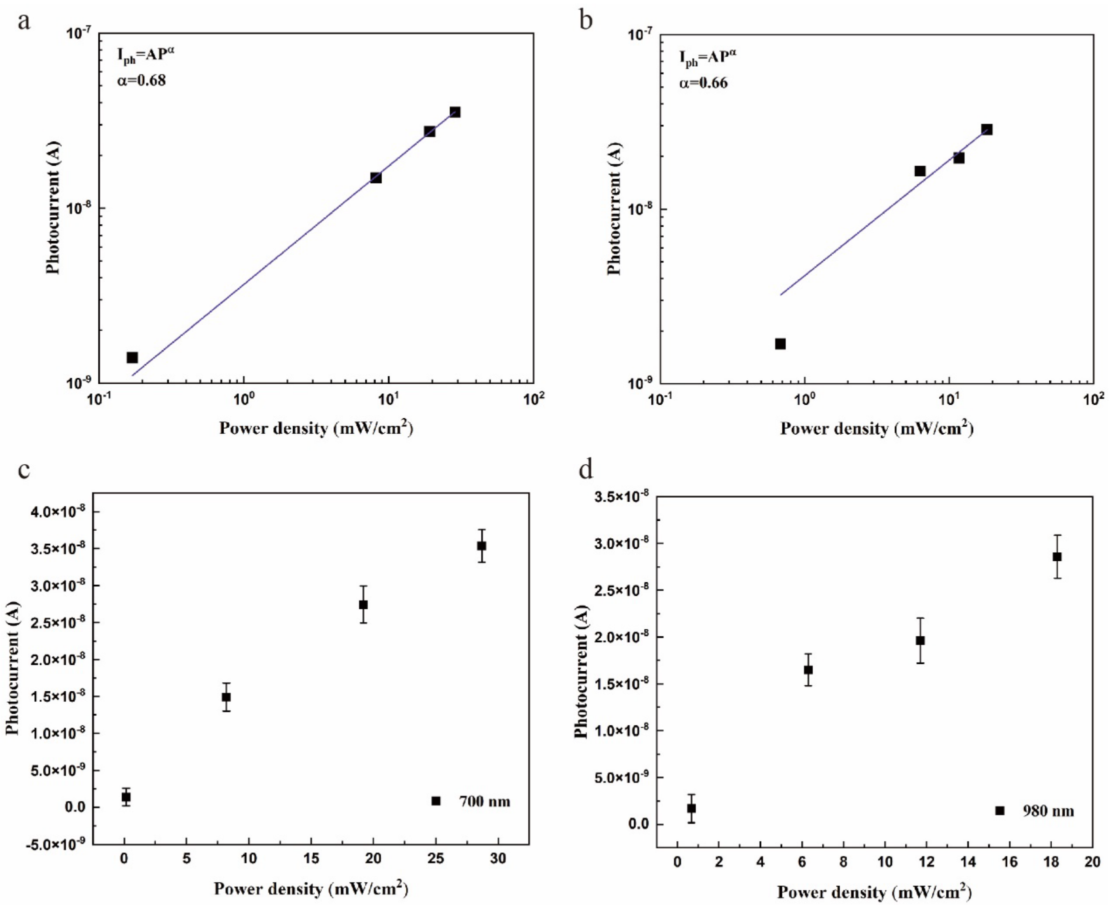


Figure S2. (a) Photocurrent as a function of power density ($V_{ds}$ = 1 V, λ = 700 nm). (b) Photocurrent as a function of power density ($V_{ds}$ = 1 V, λ = 980 nm). (c, d) Photocurrent of $PtSe_2/MoS_2$ heterostructure under 700 nm and 980 nm illumination. Error bars indicate the sample under multiple test.
